# Optimizing treatment efficacy and fertility preservation in patients undergoing hematopoietic stem cell transplantation: A narrative review of ovarian shielding with total‐body irradiation or treosulfan‐based conditioning regimens

**DOI:** 10.1002/rmb2.12648

**Published:** 2025-04-17

**Authors:** Yuji Tanaka, Tetsuro Hanada, Tsukuru Amano, Akimasa Takahashi, Mari Deguchi, Hiroyuki Yamanaka, Shunichiro Tsuji, Takashi Murakami

**Affiliations:** ^1^ Department of Obstetrics and Gynaecology Shiga University of Medical Science Otsu Shiga Japan

**Keywords:** fertility preservation, hematopoietic stem cell transplant, ovarian shielding, pediatric patients, treosulfan

## Abstract

**Background:**

Pediatric and adolescent/young adult (AYA) patients with hematologic malignancies often require hematopoietic stem cell transplantation (HSCT) using conditioning regimens that pose high risks for gonadal toxicity. Traditional protocols, including total body irradiation (TBI) and busulfan‐based regimens, can impair fertility. This review explores the potential of gonadal shielding during TBI and treosulfan‐based conditioning as strategies to optimize treatment efficacy while preserving fertility.

**Methods:**

A PubMed search up to February 2025 was performed for English, peer‐reviewed articles on hematologic malignancies, HSCT, shielding, and treosulfan. Studies on oncologic outcomes and fertility in pediatric and AYA patients were included.

**Main Findings:**

Ovarian shielding during myeloablative conditioning with TBI effectively reduces ovarian radiation exposure, resulting in improved menstrual recovery and hormone profiles. A treosulfan‐based regimen demonstrated higher antitumor activity than a reduced‐intensity busulfan‐based regimen in randomized controlled trials. In a retrospective analysis, the treosulfan‐based regimen exhibited lower gonadal toxicity than the busulfan‐based regimen, although careful attention must be paid to dosing settings of the regimens.

**Conclusion:**

Ovarian shielding during TBI and a treosulfan‐based regimen hold the potential to preserve the reproductive capacity of patients undergoing HSCT. Future clinical studies that appropriately assess both oncological outcomes and fertility are needed to validate these findings.

## 1 INTRODUCTION

Hematological malignancies are common among pediatric patients (defined as those <15 years of age) and adolescent and young adult (AYA) patients (defined as those aged 15–39 years). Together, leukemia and lymphoma account for approximately 40% of all childhood cancers. Among AYA patients, lymphoma is the most frequent type, comprising 20% of cases, while leukemia accounts for 6%. The prevalence of both lymphoma and leukemia increases with age. For instance, in the 15–19‐year age group, lymphoma is the most common (26% of cases), followed by leukemia (12%). In leukemia, survival rates generally improve with younger age, leading to a relatively high number of cancer survivors in pediatric and AYA populations.^1^, ^2^ In Japan, the annual incidence of leukemia and malignant lymphoma in patients <40 years of age is approximately 1700 and 1300 cases, respectively.^3^ Acute lymphoblastic leukemia (ALL) accounts for the largest proportion of leukemia cases, followed by acute myeloid leukemia (AML). Among malignant lymphomas, the most common subtypes include Hodgkin lymphoma (which is more frequent in Western countries than in Japan), diffuse large B‐cell lymphoma (common in both children and AYA patients), lymphoblastic lymphoma (frequent in children), and Burkitt lymphoma (also common in children).^1^, ^4^

Fertility impairment due to cancer treatment is a major concern among pediatric and AYA cancer survivors. Some patients with hematological malignancies require hematopoietic stem cell transplantation (HSCT), during which conditioning regimens consisting of either total‐body irradiation (TBI) or high‐dose alkylating agent‐based chemotherapy are administered. The risk of treatment‐related fertility impairment in patients with hematological malignancies depends on the type and cumulative dose of alkylating agents used, as well as the radiation dose delivered to the gonads. Although non‐transplant treatment protocols for hematological malignancies are generally considered to pose a low to moderate risk of gonadal toxicity, conditioning regimens for HSCT carry a uniformly high risk for both sexes.^5^, ^6^, ^7^

Current fertility preservation methods include cryopreservation of oocytes, embryos, sperm, and ovarian tissue. However, for prepubertal girls, ovarian tissue cryopreservation is the only available option, though it carries a risk of malignant cell contamination. For prepubertal boys, no established fertility preservation method currently exists. These limitations highlight the need to develop approaches beyond conventional cryopreservation, particularly given that hematological malignancies frequently affect patients even before puberty. Theoretically, if a conditioning regimen could provide non‐inferior or superior antitumor efficacy while considerably reducing the risk of fertility impairment, modifying treatment to incorporate such an approach could offer a fundamental solution. However, to the best of our knowledge, no review has explored the balance between fertility preservation and treatment efficacy in both female and male patients undergoing HSCT. Therefore, this study focuses on two aspects of fertility preservation strategies.

The first approach involves gonadal shielding during the widely used TBI plus cyclophosphamide (CY‐TBI) regimen, assessing its oncological safety and effectiveness in preserving fertility.

The second approach focuses on treosulfan. In non‐TBI conditioning protocols in Japan, the busulfan‐cyclophosphamide (BU‐CY) regimen is predominantly used as a chemotherapy‐only approach based solely on alkylating agents. Although treosulfan‐based regimens have not yet been approved in Japan, their use has grown internationally in recent years. Treosulfan is considered to have superior antitumor efficacy compared to busulfan. However, due to its relatively recent introduction—initially in Europe in January 2019, with approval in the United States in January 2025 current fertility preservation guidelines^5^, ^6^, ^7^ have yet to incorporate treosulfan. Nonetheless, several retrospective studies have demonstrated that treosulfan‐based regimens cause substantially lower gonadal toxicity than busulfan‐based regimens.^8^, ^9^, ^10^, ^11^, ^12^ This review examines the antitumor efficacy and gonadal toxicity of treosulfan in HSCT conditioning.

## 2 METHODS

Regarding article selection and inclusion criteria, we conducted a PubMed database search for articles published up to February 2025 using keywords such as “hematologic malignancies,” “lymphoma,” “leukemia,” “multiple myeloma,” “aplastic anemia,” “fertility preservation,” “hematopoietic stem cell transplant,’” “treosulfan,” “ovarian shielding,” “testicular shielding,” and “gonadal shielding.” A comprehensive literature search was performed between January and April 2025. Titles and abstracts were screened for relevance to the topic, and potentially relevant articles were selected for full‐text review. The inclusion criteria required articles to be published in English, in peer‐reviewed journals, and to specifically address either gonadal shielding or treosulfan in HSCT conditioning.

In this review, multiple abbreviations related to the therapeutic regimens for hematological malignancies are used. The commonly used abbreviations are compiled as follows: ABVD, Adriamycin, Bleomycin, Vinblastine, Dacarbazine; BCNU, Carmustine; BEACOPP, Bleomycin, Etoposide, Adriamycin, Cyclophosphamide, Vincristine, Procarbazine, Prednisone; BEAM, BCNU, Etoposide, Ara‐C (Cytarabine), Melphalan; CHOP, Cyclophosphamide, Doxorubicin, Vincristine, Prednisone; CHOPE, CHOP plus Etoposide; COPP, Cyclophosphamide, Oncovin (Vincristine), Procarbazine, Prednisone; CVP, Cyclophosphamide, Vincristine, Prednisone; DA‐EPOCH, Dose‐Adjusted Etoposide, Prednisone, Vincristine, Cyclophosphamide, and Doxorubicin; EBVP, Epirubicin, Bleomycin, Vinblastine, Prednisone; MCNU, Ranimustine; MEAM, MCNU, Etoposide, Ara‐C (Cytarabine), Melphalan; MOPP, Mechlorethamine, Vincristine, Procarbazine, Prednisone; R‐CHOP, Rituximab plus CHOP.

### 2.1 The role of haematopoietic stem cell transplantation in standard treatment for hematologic malignancies

First, we provide an overview of the major treatment strategies for hematological malignancies. Lymphomas encompass a wide range of tumor subtypes treated with a combination of chemotherapy, targeted therapy, immunotherapy, and local radiotherapy. From a chemotherapy perspective, previously used protocols—COPP, MOPP, BEACOPP (including the dose‐escalated variant), or COPP combined with ABVD—have largely been phased out. Currently, the standard regimens are ABVD for Hodgkin lymphoma and CHOP for non‐Hodgkin lymphoma, particularly diffuse large B‐cell lymphoma.^13^ These regimens include alkylating agents such as cyclophosphamide and dacarbazine, both of which have relatively high gonadotoxicity. In refractory or relapsed cases, salvage chemotherapy followed by autologous HSCT is performed if a favorable response is achieved.^14^, ^15^, ^16^ In addition, treatment for lymphoblastic lymphoma, which is common in children, typically follows an ALL‐type protocol.

The most frequently diagnosed leukemia in children is ALL. Treatment for ALL in AYA patients generally consists of induction and consolidation therapy, followed by either maintenance therapy or allogeneic HSCT. Although pediatric and adult regimens differ slightly, key agents include anthracyclines, vincristine, cytarabine, 6‐mercaptopurine, methotrexate, and L‐asparaginase. In Philadelphia chromosome‐positive cases, tyrosine kinase inhibitors play a crucial role, and cyclophosphamide‐based protocols may also be included. For AML, excluding acute promyelocytic leukemia, standard therapy consists of induction and consolidation, with risk‐adapted indications for allogeneic HSCT. Outside of allogeneic conditioning, chemotherapy primarily consists of cytarabine and anthracyclines, with alkylating agents generally not used.^17^, ^18^, ^19^, ^20^, ^21^

Although hematological malignancies often respond well to chemotherapy, HSCT remains crucial for enhancing antitumor efficacy in relapsed, refractory, or high‐risk cases.^22^ Comprehensive reviews on the principles, types, and indications of HSCT^23^, ^24^, ^25^, ^26^ highlight that the core strategy involves administering supramaximal doses of chemotherapy or TBI to destroy the patient's bone marrow and malignant cells, followed by the infusion of hematopoietic stem cells to restore hematopoiesis. In allogeneic HSCT, donor cells provide a graft‐versus‐leukemia (GVL) effect, which can potentially cure otherwise intractable diseases.

If the graft cells are derived from the patient, the procedure is referred to as autologous HSCT, thereby avoiding the risk of graft‐versus‐host disease (GVHD) and graft rejection. However, in leukemia, there is concern about the reinfusion of malignant cells.^27^ Hence, in children and AYA with lymphoma, autologous HSCT is often preferred, as the conditioning regimen alone may achieve a cure, and the risk of tumor contamination in harvested cells is relatively low. Conversely, leukemia typically requires allogeneic HSCT to harness the GVL effect. Nationally aggregated data from the Japanese Data Centre for Hematopoietic Cell Transplantation and the Japanese Society for Hematopoietic Cell Transplantation describe the disease‐specific indications for allogeneic versus autologous HSCT.

Harvested stem cells can originate from bone marrow, peripheral blood, or umbilical cord blood. Nonmalignant hematological disorders, such as aplastic anemia, may also require HSCT, although the conditioning regimens differ in both toxicity and objectives. Furthermore, HSCT is applied in certain pediatric solid tumors, such as neuroblastoma and Ewing sarcoma, as well as in primary immunodeficiency syndromes; however, these conditions typically involve different preparative regimens and are beyond the scope of this article.

### 2.2 Conditioning regimens for allogeneic transplant

Most children and AYA patients with hematological malignancies undergoing allogeneic HSCT have leukemia. The primary goal of conditioning is to eradicate malignant cells while suppressing host immunity to prevent graft rejection. In allogeneic HSCT, the treatment intensity is classified into the myeloablative conditioning (MAC) regimen and the reduced‐intensity conditioning (RIC) regimen. RIC is primarily used in older patients, those with organ dysfunction, or for second transplants.^28^, ^29^ However, for children and AYA patients, myeloablative conditioning remains the standard.

Among myeloablative regimens, CY‐TBI and BU‐CY are frequently used. TBI provides strong immunosuppression, is effective against chemotherapy‐resistant tumor cells, and ensures coverage of the central nervous system. Regimens that exclude TBI, such as BU‐CY, are preferable in centers without radiation facilities. However, all previous randomized controlled trials (RCTs) comparing CY‐TBI and BU‐CY were conducted using oral busulfan, which is no longer prescribed. Since the 2000s, three large retrospective studies—each including over 1000 patients—have been conducted following the adoption of intravenous busulfan.^30^, ^31^, ^32^ Although differences were observed in antitumor efficacy and side effects across various evaluation metrics, no consistent superiority was found. In clinical practice, the choice between CY‐TBI and BU‐CY depends on several factors. For instance, TBI is considered highly effective in ALL and other lymphoid malignancies.^33^, ^34^ In pediatric patients, BU‐CY may be preferred to avoid radiation‐induced growth impairment. Non‐sibling or human leukocyte antigen‐mismatched transplants may favor TBI due to its stronger immunosuppressive effect (Table 1).

**TABLE 1:** Comparison of conventional conditioning regimens for allogeneic transplant.

	CY‐TBI	BU‐CY
Purpose	Eradicate tumor cells using radiation plus chemotherapy Achieve potent immunosuppression	Eradicate tumor cells using chemotherapy Achieve potent immunosuppression
Advantages	Possibly more effective in lymphoid malignancies Strong immunosuppression (beneficial in mismatched transplants) Effective against chemo‐resistant tumors Reaches sanctuary sites (such as central nervous system)	Avoids radiation‐induced toxicities (growth retardation) No need for radiation equipment
Disadvantages	Gonadal toxicity Risk of growth retardation in children Requires radiation facilities	Gonadal toxicity Possibly less effective in lymphoid malignancies Busulfan‐specific toxicities (such as hepatic toxicity)

Abbreviations: BU‐CY, busulfan and cyclophosphamide; CY‐TBI, cyclophosphamide and total body irradiation.

Thus, the choice of protocol depends on multiple factors, including patient age, medical history, donor compatibility, and comorbidity risks, requiring a flexible, case‐by‐case clinical decision‐making approach.

### 2.3 Conditioning protocols for autologous transplant

Most children and young adults with hematological malignancies undergoing autologous HSCT have lymphomas. In this setting, the primary goal is tumor eradication without the need for host immunosuppression to prevent graft rejection. This allows for a less intense regimen compared to the myeloablative conditioning used in allogeneic HSCT. In Western countries, BEAM is the most commonly used regimen for lymphoma; whereas in Japan, MEAM—which substitutes MCNU (ranimustine) for BCNU—may be used.^35^

### 2.4 Risk of treatment‐induced gonadal dysfunction in patients with hematologic malignancies

Multiple guidelines have summarized the risk of fertility impairment associated with various treatment modalities for hematologic malignancies (Tables 2 and 3). However, as treosulfan‐based regimens have only recently gained widespread adoption, they have not yet been incorporated into these risk classifications.

**TABLE 2:** Risks of treatment‐related amenorrhea in female patients with hematologic malignancies.

	ESMO [5]	ESHRE [6]	*Ferti*PROTEKT [7]
High risk (>80% risk of amenorrhea)	Hematopoietic stem cell transplant (especially alkylating agent‐based MAC with CY, BU, melphalan, or TBI) RT >6 Gy to a field including the ovaries 6 to 8 cycles of escalated BEACOPP of ≥30 years	Conditioning regimens for Hematopoietic stem cell transplant with CY and/or TBI RT, including the ovaries	Hematopoietic stem cell transplant with CY‐TBI or BU‐CY RT, including the ovaries Escalated BEACOPP ≥30 years Procarbazine, Chlorambucil
Intermediate risk	6 to 8 cycles of escalated BEACOPP <30 years 6 cycles of CHOP of ≥35 years 6 cycles of DA‐EPOCH of <35 years	Alkylating agent‐based regimens (such as MOPP, BEACOPP, CHOP, and CHOPE) in patients with lymphoma	Escalated BEACOPP <30 years
Low risk (<20% risk of amenorrhoea)	2 cycles of escalated BEACOPP ABVD 6 cycles of CHOP of <35 years 6 cycles of DA‐EPOCH <35 years AML therapy (anthracycline/cytarabine) ALL therapy (multi‐agent)	Non‐alkylating agent‐based regimens (such as ABVD or EBVP) in lymphoma patients aged ≥32 years	ABVD ≥32 years 4 to 6 cycles of CHOP CVP AML therapy (anthracycline/cytarabine) ALL therapy (multi‐agent)
Very low or no risk	N/A (This risk is not set)	Non‐alkylating agent‐based regimens (such as ABVD or EBVP) in lymphoma patients aged <32 years Single‐agent methotrexate	ABVD <32 years Methotrexate, Fluorouracil, and Vincristine

Abbreviations: ABVD, adriamycin, bleomycin, vinblastine, dacarbazine; ALL, acute lymphoblastic leukemia; AML, acute myeloid leukemia; BEACOPP, bleomycin, etoposide, adriamycin, cyclophosphamide, oncovin (vincristine), procarbazine, prednisone; BU, busulfan; CHOP, cyclophosphamide, hydroxydaunorubicin (doxorubicin), oncovin (vincristine), prednisone; CHOPE, CHOP + Etoposide; CVP, cyclophosphamide, vincristine, prednisone; CY, cyclophosphamide; DA‐EPOCH, dose‐adjusted etoposide, prednisone, vincristine, cyclophosphamide, doxorubicin; EBVP, epirubicin, bleomycin, vinblastine, prednisone; ESHRE, European Society of Human Reproduction and Embryology; ESMO, European Society for Medical Oncology; FertiPROTEKT, Fertility Preservation Network in German‐Speaking Countries; MAC, myeloablative conditioning; MOPP, mechlorethamine, oncovin (vincristine), procarbazine, prednisone; RT, radiotherapy; TBI, total body irradiation.

**TABLE 3:** Risks of treatment‐related amenorrhea in male patients with hematologic malignancies.

	ESMO [5]
High risk	Radiation therapy Alkylating agents (cyclophosphamide, ifosfamide, procarbazine, cisplatin, chlorambucil, carmustine, lomustine, melphalan, thiotepa, busulfan, and mechlorethamine) with CED >5 g/m^2^ for germ cells and 20 g/m^2^ for somatic cells Conditioning chemotherapy for bone marrow transplant (busulfan and cyclophosphamide, fludarabine, and melphalan)
Intermediate risk	Alkylating agents (dacarbazine) Anthracyclines (doxorubicin, idarubicin, and daunorubicin) Mitoxantrone Antimetabolites (cytarabine and gemcitabine)
Low risk	Antimetabolites (mercaptopurine, methotrexate, and fludarabine) Tubulin‐binding agents/vinca alkaloids (vincristine and vinblastine) Topoisomerase inhibitors (etoposide) Antitumor antibiotics (bleomycin, dactinomycin, and mitomycin C)
Unknown risk	Antimetabolites (fluorouracil and thioguanine) Taxanes (paclitaxel and docetaxel) Topoisomerase inhibitors (irinotecan, topotecan, and teniposide) Immunotherapy Targeted therapies (including monoclonal antibodies and small molecules)

Abbreviations: CED, cyclophosphamide equivalent dose; ESMO, European Society for Medical Oncology.

In chemotherapy‐induced gonadal toxicity, the risk of fertility impairment varies depending on the type and cumulative dose of alkylating agents. The Cyclophosphamide Equivalent Dose (CED) is a useful metric for comparing the gonadotoxicity of different alkylators,^36^ with a CED ≥4000 mg/m^2^ being associated with an increased risk of infertility.^37^ Notably, the CED was defined around 2014,^36^ before the approval of treosulfan (in Europe in 2019), which is why treosulfan was not included in its calculations.

This review examines gonadal toxicity based on sex and disease subtype. In female patients with leukemia, treatment‐related amenorrhea has been reported in approximately 17% of cases.^13^ In Hodgkin lymphoma, amenorrhea rates are considerably higher: 55% with COPP/ABVD, 47%–56% with BEACOPP, and 40%–67% with dose‐escalated BEACOPP. However, the current standard regimen, ABVD, has an amenorrhea rate of only 3%–7%, with post‐ABVD pregnancy rates comparable to those of healthy controls.^38^, ^39^, ^40^, ^41^, ^42^, ^43^ In non‐Hodgkin lymphoma, even intensive regimens such as CHOP^44^ and mega‐CHOP^45^ exhibit low ovarian toxicity.

In male patients with lymphoma, the proportion of biological fathers posttreatment was comparable to that of healthy controls (29% vs. 32%).^46^ At 1‐year posttreatment, azoospermia rates were 0% for ABVD, 22% for CHOP, and 75% for BEACOPP.^47^ At 2 years, sperm count recovery was observed in 90% of ABVD‐treated patients and 61% of CHOP‐treated patients, with final azoospermia rates of 4% for ABVD and 89% for BEACOPP.^48^, ^49^, ^50^ Data on chemotherapy‐induced infertility in male patients with leukemia are limited (sample sizes <20),^51^, ^52^ though observed sperm count reductions tend to be transient.^13^

For patients with hematologic malignancies who do not undergo HSCT, the risk of gonadal toxicity is generally low in both sexes. However, all HSCT conditioning regimens pose a high risk of gonadal toxicity, regardless of sex.

In cases where radiation therapy is required, ovarian transposition may mitigate ovarian damage during pelvic irradiation for lymphoma.^53^, ^54^, ^55^ However, TBI—commonly used in HSCT conditioning—carries an invariably high risk of gonadal toxicity.

Thus, compared to non‐transplant treatments, HSCT substantially increases the risk of gonadal toxicity. This review focuses on patients who underwent HSCT and references Socié et al. (2003),^56^ one of the most frequently cited studies on HSCT‐related infertility, which reported extremely high gonadal toxicity with CY‐TBI and BU‐CY regimens (Table 4). More recent reports on HSCT‐related infertility^57^, ^58^, ^59^ consistently confirm that BU‐CY and CY‐TBI are among the most gonadotoxic regimens. Some evidence suggests that busulfan‐based regimens may be more gonadotoxic than TBI‐based regimens.^60^, ^61^

**TABLE 4:** Gonadal recovery rates in conventional conditioning regimens for hematopoietic stem cell transplant.

Type of SCT	Conditioning	Sex	*n*	Gonadal recovery
Allogeneic	CY	Female	43	74%
Allogeneic	CY	Female	103	54%
Allogeneic	CY	Male	109	61%
Allogeneic	BU‐CY	Female	73	1%
Allogeneic	BU‐CY	Male	146	17%
Allogeneic	TBI	Female	74	13.5% (100% < 18; 15% > 18)
Allogeneic	TBI	Female	532	10%
Allogeneic	TBI	Male	463	17.5%
Autologous	BEAM	Female	10	60%

*Note*: The BEAM regimen is widely used for autologous transplantation in lymphoma in Western countries. However, in Japan, MEAM—where BCNU is replaced by ranimustine (MCNU)—is more commonly employed. This research was originally published in Blood. Socié.^56^ © The American Society of Haematology.

Abbreviations: BEAM, carmustine, etoposide, cytarabine, melphalan; BU‐CY, busulfan, cyclophosphamide; CY, cyclophosphamide; HSCT, hematopoietic stem cell transplant; SCT, stem cell transplant; TBI, total body irradiation.

Reduced‐intensity conditioning (RIC) in allogeneic HSCT theoretically carries a lower risk of fertility impairment. Although some studies have reported a reduced risk of gonadal failure with RIC,^62^, ^63^, ^64^, ^65^, ^66^ others have found no difference in infertility risk.^9^, ^67^, ^68^, ^69^ These inconsistencies likely stem from protocol heterogeneity. Although certain patient subgroups may benefit from fertility preservation, uniformly reducing conditioning intensity solely for fertility preservation is not recommended unless necessitated by organ impairment.

Notably, in the report by Socié et al. (2003),^56^ cyclophosphamide monotherapy, primarily used for benign conditions such as aplastic anemia, has shown a 50%–70% gonadal recovery rate. In female patients with lymphoma undergoing autologous HSCT, Socié et al. reported high gonadal recovery rates with the BEAM regimen, with subsequent studies reporting menstrual resumption rates of 68%^70^ and 63%.^71^ However, male patients appear to be at a higher risk of gonadal toxicity post‐HSCT. Some studies indicate that 12 out of 18 patients became azoospermic,^72^ and in some cohorts, no recovery was observed.^73^ Even the largest systematic review (2022) on male reproductive function after HSCT,^74^ detailed data on patients limited solely to lymphoma patients undergoing HSCT are not presented.

### 2.5 Risk factors for treatment‐induced gonadal dysfunction in patients with hematologic malignancies

A key clinical question is whether malignancies themselves contribute to reduced fertility. In women, a meta‐analysis of ovarian reserve before gonadotoxic treatment found that while breast cancer does not appear to affect fertility, hematologic malignancies do.^75^ Several other studies have similarly suggested that simply having a hematologic malignancy may be a risk factor for reduced fertility in women.^76^, ^77^, ^78^ In men, baseline fertility is also diminished in patients with hematologic malignancies.^79^

Age is another important factor. In women, baseline fertility naturally declines with age, making the patient's age at the start of treatment a critical predictor of posttreatment fertility.^57^ Some guidelines even classify the risk of infertility differently depending on the patient's age. In contrast, regarding testicular toxicity, reports are suggesting that age is not a major risk factor.^80^, ^81^, ^82^, ^83^

Among recipients of allogeneic HSCT, chronic GVHD may further increase the risk factor of infertility. In one study of male patients, only 2 of 11 men with chronic GVHD had detectable sperm 2–20 years posttransplant (median, 9 years) compared to 16 of 28 men without GVHD.^58^ In women, studies have shown that 1–2 years posttransplant, those with chronic GVHD have considerably smaller ovaries and uteri.^84^ Genital GVHD is common in female patients,^85^, ^86^, ^87^, ^88^ with 76% experiencing vulvovaginal atrophy and 92% reporting sexual dysfunction. Statistically significant reductions in self‐reported female sexual function indices—adversely affecting couple and marital relationships—have been documented.^89^ Experimental models have further demonstrated that GVHD impairs ovarian function.^90^ Notably, the incidence of genital GVHD is higher in women than in men (22% vs. 5%).^86^ These findings highlight the importance of effective GVHD prevention and management in allogeneic transplant recipients, particularly in pediatric and AYA patients with leukemia, to minimize fertility impairment.

Overall, HSCT recipients face a higher risk of fertility impairment than those undergoing autologous transplant. In a study involving lymphoma patients (median age: 25 years), differences in conditioning regimen intensity and immune regulation resulted in poorer menstrual recovery following allogeneic transplants compared to autologous transplants.^84^ Among female patients with lymphoma, autologous HSCT had a menstrual recovery rate of 63%.^71^ Regarding obstetric complications, HSCT is associated with an increased incidence, with allogeneic HSCT resulting in more complications than autologous HSCT.^91^, ^92^ These findings emphasize the need for improved fertility preservation strategies in standard allogeneic conditioning regimens (CY‐TBI and BU‐CY) for pediatric and AYA patients.

Furthermore, psychological factors—such as depression, sexual accessibility, and the desire to have children—can influence fertility outcomes and impact quality of life (QOL).^93^, ^94^, ^95^, ^96^, ^97^, ^98^

Although detailed reviews on perinatal outcomes after HSCT exist, they are beyond the scope of this review.^99^

### 2.6 Fertility preservation challenges unique to patients with hematologic malignancies and the advantages of modifying primary treatment

A review summarizing fertility preservation techniques in patients with hematologic malignancies has been published^100^ (see Tables 5 and 6). Conventional fertility preservation for women typically involves oocyte or embryo cryopreservation for postpubertal patients, a method supported by high levels of evidence. The introduction of random‐start ovarian stimulation has reduced treatment delays to 2–3 weeks.^101^ However, some patients with hematological malignancies experience rapid tumor progression, leaving insufficient time for oocyte or embryo cryopreservation. In such cases, ovarian tissue cryopreservation is the only option. In addition, ovarian tissue cryopreservation is the sole fertility preservation method available for prepubertal females. Consequently, the demand for ovarian tissue cryopreservation is higher in female patients with hematologic malignancies than in those with other cancers, with patients with lymphoma comprising approximately one third of all ovarian tissue transplant cases.^102^ Despite its potential benefits, ovarian tissue cryopreservation carries a risk of malignant cell contamination.^103^, ^104^, ^105^, ^106^, ^107^ Although some studies suggest that viable leukemic cells may not persist in cryopreserved ovarian tissue,^108^, ^109^ molecular analyses have detected malignant cells in approximately half of the ovarian tissue samples from patients with leukemia.^110^ Although reports indicate that, during marrow remission, the risk of tumor cell contamination is low,^108^, ^111^ these findings are over 10 years old, and it remains uncertain whether the findings are reproducible using modern detection methods. The risk of tumor cell contamination in lymphomas has also been discussed^102^, ^112^, ^113^; however, it is considered relatively low.^103^ Several detection methods have been proposed, including immunohistochemical analysis, polymerase chain reaction (PCR), multicolor flow cytometry, and xenografting combined with next‐generation sequencing.^114^, ^115^, ^116^, ^117^ However, challenges such as variability between different sections of the same ovary persist,^115^ and no optimal detection method has been established. Several investigational approaches aim to reduce the risk of tumor cell contamination in ovarian tissue, including in vitro maturation of oocytes,^118^, ^119^ the development of an “artificial ovary,”^120^ and methods for removing tumor cells from ovarian cortex fragments.^121^, ^122^, ^123^ However, these strategies remain in the preclinical stage. In addition, in patients who undergo unilateral oophorectomy for ovarian tissue cryopreservation, the incidence of early ovarian failure is substantially higher in those with hematologic malignancies than in patients with breast cancer (87% vs. 9%), even among those who did not receive HSCT.^124^ This underscores the risk of permanent fertility loss if cryopreserved ovarian tissue is contaminated with MRD and cannot be transplanted.

**TABLE 5:** Fertility preservation options in female patients with hematologic malignancies.

	Embryo cryopreservation	Oocyte cryopreservation	Ovarian tissue cryopreservation	GnRH analogue	Modification of clinical care
Adaptable generation	Postpubertal	Postpubertal	Prepuberty Postpubertal	Postpubertal	Prepuberty Postpubertal
Evidence	Standard option	Standard option	Clinical option	Clinical option	Accumulating evidence
Advantages	Established method No tumor contamination	Established method No partner required No tumor contamination	No treatment delays Many follicles preserved Natural conception No partner required Only prepubertal clinical option	No treatment delays High accessibility Low cost Low invasion Natural conception No tumor contamination No partner required Hypomenorrhea	Prepubertal option No treatment delays High accessibility Low cost Low invasion Natural conception No tumor contamination No partner required Prepuberty option
Technical disadvantages	Treatment delay Requires a male partner Embryo storage limited Not for prepubertal use	Treatment delay Oocyte storage limited Not for prepubertal use	Tumor contamination Requires surgery Not a standard option	Inconsistent evidence Hot flashes Sexual dysfunction Osteoporosis Not for prepubertal use	Weak evidence
Non‐technical disadvantages	Cost issue Moderate accessibility issue	Cost issue Moderate accessibility issue	Cost issue High accessibility issue		

Abbreviation: GnRH, gonadotropin‐releasing hormone.

**TABLE 6:** Fertility preservation options in male patients with hematologic malignancies.

	Sperm cryopreservation	Testicular tissue cryopreservation	Modification of clinical care
Adaptable generation	Postpubertal	Prepuberty	Prepuberty Postpubertal
Evidence	Standard option	No clinical evidence	Accumulating evidence
Advantages	Established method	Potential to preserve reproductive function even in prepubescent boys (Research is ongoing to obtain mature sperm from immature spermatogonia)	Prepubertal option High accessibility Low cost Low invasion No tumor contamination
Technical disadvantages	In cases of erectile dysfunction, vibratory stimulation, electroejaculation, or testicular sperm extraction may be required	Tumor contamination Requires surgery No clinical evidence	Weak evidence
Nontechnical disadvantages	Cost issue Moderate accessibility issue	Cost issue	

For male patients, sperm cryopreservation in postpubertal individuals is supported by strong evidence.^125^, ^126^, ^127^ Even when spontaneous ejaculation is not possible, alternative techniques such as electrical stimulation^128^, ^129^ or testicular sperm extraction (TESE)^127^, ^130^, ^131^, ^132^, ^133^ can facilitate sperm preservation. However, testicular tissue cryopreservation is not yet clinically applicable for prepubertal boys.^134^ This gap highlights a critical challenge in fertility preservation for pediatric patients with hematological malignancies.

Pharmacologic approaches have been explored, but with limited success. Although GnRH analogs effectively reduce menstrual bleeding in patients undergoing HSCT, they do not appear to improve the rate of premature ovarian failure.^135^, ^136^, ^137^

An alternative strategy involves modifying primary treatment to reduce gonadal toxicity while maintaining antitumor efficacy. This approach offers several advantages: It does not delay treatment, is cost‐effective, and improves international accessibility; eliminates the need for surgical intervention; allows for natural conception; avoids the risk of malignant cell contamination; and does not require a partner. Most importantly, it can be applied to prepubertal patients. However, the treatment strategy for malignant tumors must prioritize antitumor efficacy. Ideally, an alternative regimen should be at least non‐inferior and preferably superior in effectiveness.

### 2.7 Gonadal shielding in CY‐TBI: Fertility preservation efficacy and oncologic safety

The severity of TBI‐induced ovarian toxicity depends on the radiation dose.^138^ Exposure to as little as 2 Gy can result in a 50% loss of oocytes.^139^ The radiation dose that causes immediate ovarian failure in 97.5% of patients has been reported as follows: 20.3 Gy at birth, 18.4 Gy at 10 years, 16.5 Gy at 20 years, and 14.3 Gy at 30 years.^140^ However, since TBI is typically administered in combination with cyclophosphamide, and ovarian function may already be compromised by prior chemotherapy, even lower doses could induce ovarian failure. Although some reports suggest that reducing the TBI dose may help preserve fertility,^141^ the primary goal of HSCT conditioning is complete tumor eradication. Any modification that diminishes antitumor efficacy, even slightly, is not recommended.

Conversely, a conditioning regimen incorporating TBI with gonadal shielding theoretically enables fertility preservation without reducing treatment intensity in target organs such as the bone marrow. This approach represents a promising strategy for female patients wishing to preserve fertility. At TBI, lung shielding using metal is sometimes used to reduce lung toxicity.^142^, ^143^, ^144^ Similarly, strategies for protecting other critical organs have been explored, including renal shielding^145^ and lens shielding for cataract prevention.^146^ As part of these protective measures, gonadal shielding for fertility preservation has been investigated at several institutions.

Two ovarian shielding techniques have been reported by two Japanese groups: the University of Tokyo Hospital^147^, ^148^ and Jichi Medical University (in collaboration with Jichi Medical University Saitama Medical Centre^149^, ^150^, ^151^, ^152^, ^153^). These techniques include the long‐source‐axis distance method and the moving‐table method. No significant difference in fertility preservation outcomes has been observed between the two methods (*p* = 0.85),^153^ suggesting that the choice of method should be based on available radiation equipment. Furthermore, intensity‐modulated radiation therapy using helical tomotherapy, which has been increasingly adopted to reduce radiation toxicity, has demonstrated the potential to enable safer TBI treatment^154^, ^155^, ^156^, ^157^ and may be considered in combination with gonadal shielding. Metallic ovarian shielding reportedly reduces the actual ovarian radiation dose from approximately 12 Gy to 2–3 Gy,^158^ a level unlikely to result in permanent infertility.^159^ Moreover, MRI studies have shown that the safe volume encompassing 95% of ovarian displacement is 11 cm for the left ovary and 25 cm for the right ovary,^160^ providing essential information for ovarian shielding planning.

Menstrual resumption rates exceeded 50%, with higher rates observed over the long term. Notably, researchers from Jichi Medical University and Jichi Medical University Saitama Medical Centre have consistently reported multifaceted outcomes.^149^, ^150^, ^151^, ^152^, ^153^ Although these studies lacked control groups, earlier reports indicate that the menstrual resumption rate in patients receiving TBI is only 10%–13%,^56^ suggesting that ovarian shielding substantially enhances fertility preservation. Major differences in AMH levels were also observed.

An additional benefit of ovarian shielding is the reduction in radiation exposure to the uterus. Studies have shown that ovarian shielding reduces the uterine radiation dose to 60% (approximately 7 Gy), and there have been two reported cases of live births following shielding.^149^ Recent reviews have emphasized the risks of uterine toxicity caused by radiation.^161^ A history of TBI is associated with increased risks of preterm birth and low birth weight compared to the general population.^92^ TBI has also been associated with higher rates of spontaneous abortion (38% vs 4%) and preterm delivery (63% vs 18%) than chemotherapy‐only regimens.^61^ Women with a history of pelvic irradiation have a considerably higher risk of preterm birth following oocyte donation than those without prior radiation exposure.^162^ Uterine toxicity is dose‐dependent: doses >5 Gy increase the risk of low birth weight and preterm delivery,^163^ doses >10 Gy increase the risk of stillbirth and neonatal mortality,^164^ and doses between 14 and 30 Gy can lead to reduced uterine volume, decreased elasticity of the myometrium, and damage to uterine vasculature^165^ Given that the standard myeloablative TBI protocol delivers approximately 12–14.4 Gy to the uterus, reducing the uterine dose through ovarian shielding may not only protect ovarian function but also help preserve uterine function for future pregnancies.

Concerns have been raised that ovarian shielding might reduce antitumor efficacy. However, from the perspective of ensuring adequate radiation dose to the bone marrow, data have shown that even with ovarian shielding, the pelvic bone dose remains above the minimum threshold of 9.9 Gy^166^ required for tumor control. In a study involving 20 cases with ovarian shielding, it was emphasized that all five relapses occurred as hematologic relapses, with no extramedullary recurrences observed, and that no significant differences in radiation dose were found between relapsed and non‐relapsed patients.^150^ Regarding the question of which patients might have a higher relapse risk following ovarian shielding, an observational study of 19 patients reported that among patients with AML and ALL, 2 out of 14 relapsed, whereas both patients with acute undifferentiated leukemia (AUL) and blastic plasmacytoid dendritic cell neoplasm (BPDCN) experienced relapse. Additionally, relapse was observed in patients with acute leukemia who did not respond to induction therapy and in patients with myelodysplastic syndrome (MDS) featuring hyperproliferative blasts.^151^ These clinical data regarding relapses may be useful for guiding patient selection for ovarian shielding. In TBI with ovarian shielding, a metal block is placed over the ovary so that it does not shield the pelvis. However, in some cases, part of the pelvis could be shielded. Even in such cases, based on a report from Seattle,^167^ of a HSCT with TBI 2Gy RIC conditioning regimen, it may not increase relapses in standard‐risk patients. Moreover, the risk of recurrence may be further reduced by combining high‐dose cyclophosphamide or cytarabine. Therefore, it is acceptable for a portion of the pelvis to be slightly blocked. In summary, these findings suggest that, with appropriate case selection based on specific criteria, ovarian shielding may not compromise oncological outcomes; however, further clinical studies are warranted.

It is important to note that there are no published reports on ovarian shielding in pediatric patients. Therefore, the feasibility of ovarian shielding in children with small uteri and ovaries requires further investigation. Paradoxically, because current ovarian shielding indications are limited to postpubertal females, the major limitation of TBI—growth impairment—is not a contraindication for choosing TBI in these patients.

Notably, a decision analysis of the risks and benefits of ovarian protection in female patients receiving TBI has also been reported.^168^

Testicular shielding, however, is not recommended. Although some studies suggest that testicular shielding during TBI can preserve fertility, most studies have focused on benign conditions, such as aplastic anemia,^169^ sickle cell disease, hereditary spherocytosis,^170^ and inherited metabolic or blood disorders.^171^ In patients with leukemia, standard TBI doses (12–13.2 Gy) have historically been associated with high testicular relapse rates. Studies by Shank et al. in the 1980s and 1990s demonstrated that testicular boost therapy considerably reduced relapse rates in male patients with leukemia.^172^ Consequently, many centers administer approximately 4 Gy of testicular boost, even in children, to enhance treatment efficacy.^173^ Although a 2022 single‐centre study reported no correlation between testicular boost irradiation and oncological outcomes,^174^ no equivalent studies have evaluated the safety of testicular shielding, unlike ovarian shielding. In addressing the clinical question of whether adding boost irradiation to the testes in addition to TBI increases testicular toxicity, one report found it was associated with an increased risk of azoospermia (all patients were azoospermic).^81^ However, other reports have found no significant differences in testicular volume or follicle‐stimulating hormone (FSH) levels.^175^, ^176^

### 2.8 The importance of alkylating agents in standard treatment for patients with hematologic malignancies

As described above, in pediatric and AYA patients with hematologic malignancies, alkylating agents play a crucial role in first‐line standard regimens for malignant lymphoma and myeloablative conditioning regimens for allogeneic HSCT in patients with leukemia. These agents are essential components of treatment protocols.

Due to their high gonadotoxicity, efforts in other medical fields have explored strategies to omit alkylating agents for fertility preservation. For instance, in systemic lupus erythematosus, approximately half the pediatric patients were previously treated with cyclophosphamide as an immunosuppressant, with approximately 30% developing ovarian failure.^177^ However, mycophenolate mofetil, which acts via a different mechanism, demonstrated equivalent efficacy while reducing the risk of amenorrhea to one sixth. This led to its adoption in clinical practice in the 2010s.^178^ Similarly, in breast cancer, a phase III randomized controlled trial tested the omission of cyclophosphamide using non‐inferiority criteria for both fertility preservation and antitumor efficacy, yielding favorable results.^179^

However, in hematologic malignancies, omitting alkylating agents as first‐line chemotherapy is not acceptable. Attempts to exclude dacarbazine from Hodgkin's lymphoma regimens have been unsuccessful.^180^, ^181^ Comparisons of alkylator‐ and anthracycline‐based regimens in pediatric Hodgkin's lymphoma have reinforced the effectiveness of alkylator‐based approaches.^182^ In HSCT conditioning, alkylating agents, such as busulfan (BU), cyclophosphamide (CY), melphalan (MEL), carmustine (BCNU), and ranimustine (MCNU) are even more critical than those used in first‐line chemotherapy. Their advantages include high penetrance into the bone marrow, strong immunosuppressive properties, and cell‐cycle–independent mechanisms of action.^183^ Unlike antimetabolites (such as cytarabine [Ara‐C] or fludarabine [FLU]), anthracyclines, or etoposide (ETP), alkylating agents exhibit a linear, non‐saturable relationship between concentration and antitumor effects.^184^ Therefore, regardless of the type of HSCT (allogeneic or autologous) or underlying disease, almost all conditioning regimens must include an alkylating agent.

However, the degree of fertility impairment varies among alkylating agents. The CED provides a quantitative method to compare their gonadotoxicity. Although dacarbazine has a somewhat different mechanism of action from representative alkylating agents such as cyclophosphamide—and is therefore not incorporated into the CED calculation formula—in regimen comparisons, dacarbazine used in the standard ABVD regimen for malignant lymphoma in first‐line chemotherapy exhibits lower gonadal toxicity than cyclophosphamide and procarbazine used in BEACOPP.^41^, ^185^, ^186^, ^187^ In addition, an R‐CHOP (which includes CY) has been associated with a higher risk of fertility impairment than ABVD (which includes dacarbazine).^188^ In HSCT conditioning, cyclophosphamide monotherapy resulted in substantially lower fertility impairment than BU‐CY, highlighting busulfan's severe gonadotoxicity (Table 4). This study focuses on treosulfan, which has not yet been approved in Japan but has gained favor in Europe since its EMA approval in 2019. It received FDA approval in January 2025. We evaluated treosulfan's antitumor efficacy and fertility‐preserving potential.

### 2.9 Fertility preservation by replacing Busulfan‐based regimen with a Treosulfan‐based regimen

The following sections evaluate treosulfan with busulfan‐based regimens:

A 2024 multicenter retrospective study involving 20 pediatric transplant centers under the Associazione Italiana Ematologia Oncologia Pediatrica^8^ analyzed late toxicities, including gonadal toxicity, in 521 patients (ages 0–25) with ALL, AML, or MDS who underwent allogeneic transplant. Male patients were assessed for gonadal toxicity via physical examination for delayed or precocious puberty and abnormal hormone levels (including inhibin B). Female patients were screened for delayed or precocious puberty and amenorrhea with abnormal hormone levels (including AMH). Gonadal toxicity was observed in 38% of busulfan‐treated patients versus 10% of treosulfan‐treated patients (*p* = 0.02). Among postpubertal patients (*n* = 197; median age at last follow‐up: 21 years), a statistically significant difference was observed (37% vs 17%, *p* = 0.03). Multivariate analysis indicated that treosulfan use reduced the relative risk (RR) of gonadal toxicity to 0.51 (95% CI, 0.34–0.76; *p* = 0.0009). Notably, male patients exhibited lower gonadal toxicity (RR = 0.37), and younger patients were more protected (RR = 0.46). Although no significant differences were observed in non‐gonadal late toxicities, treosulfan showed trends toward lower incidences of growth impairment, thyroid dysfunction, and secondary malignancies. The overall rate of late toxicities was significantly lower with treosulfan (34% vs. 20%, *p* = 0.01). This multicenter study, which included only malignant diseases and used robust evaluation methods incorporating AMH and inhibin B levels, provides high‐level evidence.

The single‐center retrospective study by van der Stoep et al.^9^ compared busulfan‐based and treosulfan‐based regimens in 88 patients (both male and female), defining gonadal toxicity based on FSH and luteinizing hormone (LH) levels. Permanent gonadal toxicity was observed in 55% of the busulfan group versus 13% of the treosulfan group. Even at lower treosulfan doses, gonadal toxicity did not differ significantly, suggesting that dose reduction may not be necessary for fertility preservation. Although this study focused on nonmalignant diseases, the data are promising.^9^

A study by de Kloet et al.^10^ examined 156 pediatric patients: Ovarian dysfunction (defined by FSH and LH levels) was 94% with busulfan‐based regimens versus 33% with treosulfan‐based regimens, while testicular dysfunction was 46% versus 14%, respectively. The hazard ratio for ovarian dysfunction in the busulfan group was 10.6 (95% CI, 2.2–52.7; *p* = 0.004).

Leiper et al.^11^ conducted a retrospective study in 66 patients, including those with malignant diseases, assessing AMH (females) and inhibin B (males). Significant differences favored treosulfan treatment.

Faraci et al.^12^ performed a multicenter study involving 137 patients, including those with malignant cases, to evaluate spontaneous puberty achievement in prepubertal children and spontaneous menarche in both prepubertal and postpubertal girls, with quantitative hormone level assessments. The results were as follows: For spontaneous puberty in prepubertal children, 38% versus 100% in females and 85% versus 100% in males; for spontaneous menarche, 10% versus 100% in prepubertal girls and 65% versus 75% in postpubertal girls. Significant differences in hormone levels (excluding AMH and inhibin B) were noted.^12^

Across multiple studies, treosulfan‐based regimens consistently demonstrate lower gonadal toxicity than busulfan‐based regimens, irrespective of malignancy status, age, or sex. Various evaluation methods—including assessments of pubertal onset, menstrual resumption, hormone levels, and combined clinical‐hormonal criteria—confirm treosulfan's efficacy in preserving fertility. The comparison of the gonadal toxicity of treosulfan‐ and busulfan‐based regimens is summarized in Table 7.

**TABLE 7:** Gonadal toxicity of treosulfan versus busulfan‐based regimens.

Publish (year)	Study type	*n*	Primary disease	Chemotherapy dose	Result (BU vs. Treo)	Reference number
2024	Multicenter, retrospective	521	Malignant	BU: 12.8–16 mg/kg Treo: 30–42 g/m^2^	All groups: Gonadal toxicity was 38% vs. 10% (*p* = 0.02), RR was 0.51 (95% CI 0.34–0.76, *p* < 0.001) Postpubertal group (*n* = 197): Gonadal toxicity was 37% vs. 17% (*p* = 0.03)	^8^
2023	Single center, retrospective	88	Nonmalignant	BU: High^a^ 66% vs. Low^b^ 34% Treo: High^a^ 26% vs. Low^b^ 74%	Gonadal dysfunction: 63% vs. 28% Gonadal dysfunction (permanent): 55% vs. 13%	^9^
2022	Single center, retrospective	156 (children only)	Nonmalignant	Both regimens used a myeloablative conditioning regimen	Ovarian dysfunction: 94% vs. 33% Testicular dysfunction: 46% vs. 14%	^10^
2020	Retrospective	66	Malignant, nonmalignant	BU: 14 mg/kg (*n* = 3), 16 mg/kg (*n* = 16), 20 mg/kg (*n* = 6) Treo: 36 mg/kg (*n* = 16), 42 mg/kg (*n* = 24), and unknown (*n* = 1)	Female mean AMH: 0.11 μg/L vs. 1.59 μg/L (*p* < 0.001) Male Inhibin B SDS ‐1.23±1.41 vs. ‐0.506±2.112 (*p* < 0.05)	^11^
2019	Multicenter, retrospective	137	Malignant, nonmalignant	BU: ≥8 mg/kg Treo: 36–42 g/m^2^	Spontaneous puberty achievement in prepubertal children Female: 38% vs. 100% Male: 85% vs. 100% Spontaneous menarche in Prepubertal and Postpubertal Girls Prepubertal: 10% vs. 100% Postpubertal: 65% vs. 75% Hormone levels Female: median of FSH is 78 vs. 7 (*p* = 0.003) Male: median of LH is 5.6 vs. 5.5 (*p* = 0.03)	^12^

Abbreviations: AMH, anti‐müllerian hormone; BU, busulfan; CI, confidence interval; FSH, follicle‐stimulating hormone; LH, luteinizing hormone; RR, relative risk; SDS, standard deviation scores; Treo, treosulfan.

^a^ ≥70 mg*h/L for busulfan, ≥1750 mg*h/L for treosulfan.

^b^ <70 mg*h/L for busulfan, <1750 mg*h/L for treosulfan.

However, a limitation of these retrospective studies is that the doses of treosulfan and busulfan vary across investigations. Therefore, it underscores the importance of including gonadal toxicity as a key end point in future Phase III trials comparing treosulfan‐ and busulfan‐based myeloablative regimens.

### 2.10 Antitumor efficacy and systemic toxicity of Treosulfan‐based regimens

Treosulfan‐based regimens have demonstrated superior antitumor efficacy to those of busulfan‐based regimens. One of the most impactful randomized controlled trials investigating this difference is the MC‐FludT.14/L Trial II (NCT00822393), with its final analysis published in 2022.^189^

This phase III study included 570 patients, all of whom were adults (18–70 years) diagnosed with AML or MDS, with a Karnofsky performance status ≥60%, and either age ≥50 years or a hematopoietic cell transplantation comorbidity index (HCTCI) score >2. Patients were randomized into treosulfan (*n* = 280) or busulfan (*n* = 290) conditioning arms. The primary end point, overall survival (OS), was significantly improved in the treosulfan group compared to the busulfan group, with a hazard ratio of 0.67 (95% CI: 0.51, 0.90). Subgroup analyses showed similar trends for AML (HR: 0.73, 95% CI: 0.51, 1.06) and MDS (HR: 0.64, 95% CI: 0.40, 1.02). Since its introduction in Europe in 2019, treosulfan‐based regimens have considerably replaced busulfan‐based regimens, and based on these findings, the FDA approved treosulfan in January 2025 for use in allogeneic HSCT conditioning for both adult and pediatric patients (age ≥1 year) with AML or MDS. However, it is important to note that the busulfan regimen used in the study was not myeloablative but rather a reduced‐intensity conditioning regimen (treosulfan 30 g/m^2^‐based regimen versus busulfan 6.4 mg/kg‐based regimen). Typically, in patients with hematological malignancies, a myeloablative busulfan regimen (approximately 12.8 mg/kg) is used when tolerated. In the absence of studies comparing transplant outcomes with myeloablative‐dose busulfan and treosulfan, these findings should be interpreted in the context of reduced‐intensity conditioning.

In addition, a randomized phase II trial comparing treosulfan‐ and busulfan‐based regimens in nonmalignant diseases demonstrated improved treatment‐related mortality, overall survival, and transplant‐related mortality with treosulfan.^190^ Treosulfan also exhibits lower systemic toxicity, often earning it the classification of a “low‐toxicity regimen.”^11^ In contrast, busulfan is associated with a higher incidence of severe adverse effects, including veno‐occlusive disease (VOD), interstitial pneumonia, hemorrhagic cystitis, permanent alopecia, convulsions, and mucositis. Treosulfan, however, has been reported to cause considerably lower extramedullary toxicity.^191^

### 2.11 Methods for evaluating the risk of fertility decline in patients with hematologic malignancies

By 2023, ASCO recommended that clinical trials focusing on cure or primary prevention in premenopausal women should incorporate ovarian toxicity assessments.^192^ Evaluating ovarian function involves collecting baseline data, followed by assessments every 6–12 months during treatment, at the end of therapy, and 12–24 months posttreatment. Clinical indicators of ovarian function, along with biomarkers such as anti‐Müllerian hormone, follicle‐stimulating hormone, and estradiol, should be measured. Long‐term monitoring of AMH posttreatment is particularly emphasized, as some studies suggest AMH levels may recover over time after HSCT.^151^ If biomarker evaluation is not feasible, clinical indicators such as menstruation, pregnancy, childbirth, and relevant confounders (such as a history of hysterectomy, bilateral oophorectomy, attempts at conception, use of hormonal contraceptives, endocrine therapy, GnRH agonists, or assisted reproductive technologies) should be documented. In men, fertility assessment should include semen analysis, paternity outcomes, and endocrine markers (FSH, LH, and testosterone).^193^

However, the risk of fertility decline in women with hematologic malignancies has often been evaluated based on subjective markers, such as menstrual recovery rates. Future randomized controlled trials should incorporate more precise assessment methods to generate more reliable data on fertility outcomes.

### 2.12 Future prospects for modified conditioning strategies to preserve fertility in hematopoietic stem cell transplant

The primary approach for fertility preservation remains the cryopreservation of oocytes, embryos, ovarian tissue, or sperm. When these methods are feasible, modifications to primary cancer treatment for fertility preservation should be approached cautiously. The goal in pediatric and AYA patients with hematologic malignancies is to maximize therapeutic efficacy. For instance, in pediatric ALL, TBI is commonly used. Since no fertility preservation options currently exist for prepubertal males—where cryopreservation nor gonadal shielding are inapplicable—choosing a chemotherapy‐based regimen instead of TBI might theoretically preserve fertility. However, findings from the FORUM trial (a 2020 phase III study in pediatric ALL) revealed that chemotherapy‐based conditioning (including a treosulfan did not meet non‐inferiority criteria compared to TBI plus etoposide, leading to early trial termination).^194^ Therefore, it is crucial that, once the primary treatment team presents oncologically safe treatment protocol options, the fertility preservation team evaluates the associated risk of fertility decline within those options.

Future large‐scale observational studies should investigate the oncologic safety of ovarian shielding in CY‐TBI regimens, along with its efficacy in fertility preservation. Due to ethical constraints, randomized controlled trials that directly compare ovarian shielding with non‐shielding are difficult to conduct in patients desiring fertility preservation. However, an alternative approach—such as a prospective clinical trial in which ovarian shielding is performed, with fertility preservation as the primary end point and antitumor efficacy assessed using an external control cohort—may provide valuable insights. This strategy was successfully employed in the POSITIVE trial,^195^ which evaluated treatment modifications in patients with breast cancer desiring future pregnancies. In addition, large‐scale retrospective studies may further clarify the impact of ovarian shielding on both fertility outcomes and treatment efficacy.

Retrospective analyses comparing treosulfan‐based and busulfan‐based regimens suggest that treosulfan may be associated with lower gonadal toxicity. However, it is important to note that the clinical trial underpinning the substitution of busulfan with treosulfan—the MC‐FludT.14/L Trial II—employed reduced‐intensity conditioning doses. When these findings are integrated, they potentially support the use of treosulfan‐based conditioning—with a secondary expectation of reduced gonadal toxicity—only in patients for whom reduced‐intensity conditioning is acceptable or necessary due to organ impairment.

Nonetheless, caution is warranted in interpreting these retrospective analyses evaluating gonadal toxicity, as the doses used vary considerably and they are subject to inherent biases related to patient selection and fluctuations in treatment intensity. To overcome these limitations, it would be desirable to perform post hoc analyses of gonadal toxicity in previously published randomized trials (e.g., the MC‐FludT.14/L Trial II).

Most importantly, no studies have compared transplant outcomes between myeloablative‐dose busulfan and treosulfan. Conversely, this implies that in future randomized controlled trials comparing myeloablative‐dose busulfan with treosulfan, assessing gonadal toxicity as a predefined end point at the trial's inception may not only demonstrate the superiority or non‐inferiority of hematopoietic stem cell transplant outcomes but also prove a lower gonadal toxicity profile.

## 3 CONCLUSION

Although the level of evidence is not high, retrospective studies suggest that ovarian shielding during TBI and treosulfan‐based conditioning (instead of BU‐CY) may preserve reproductive capacity without compromising oncologic efficacy. However, the pivotal studies supporting treosulfan employed reduced‐intensity conditioning doses, and no direct comparisons have been made with myeloablative‐dose busulfan. Thus, these findings primarily support treosulfan‐based conditioning in patients for whom reduced‐intensity conditioning is acceptable or required due to organ impairment. Future studies—particularly large‐scale retrospective analyses, prospective trials, and post hoc evaluations of existing randomized trials—are needed to validate these findings. Moreover, future randomized trials comparing myeloablative‐dose busulfan with treosulfan should predefine gonadal toxicity as an end point to better establish the reproductive benefits of treosulfan.

## CONFLICT OF INTEREST STATEMENT

The authors declare no conflict of interest.

## Data Availability

Data sharing is not applicable to this article as no new data were created or analyzed in this study.
